# Targeted genome mining for microbial antitumor agents acting through DNA intercalation

**DOI:** 10.1016/j.synbio.2023.07.003

**Published:** 2023-07-22

**Authors:** Zhijie Zhao, Guiyun Zhao, Yi Chai, Wei Li, Kaihui Song, Wenbin Zhao, Hairong Li, Miaolian Wu, Zhan Zhou, Yi-Ling Du

**Affiliations:** a The Fourth Affiliated Hospital and Department of Microbiology, School of Medicine, Zhejiang University, Hangzhou, 310058, China; b Yong Loo Lin School of Medicine, National University of Singapore, Singapore; c Innovation Institute for Artificial Intelligence in Medicine, College of Pharmaceutical Sciences, Zhejiang University, Hangzhou, 310058, China; d The Fourth Affiliated Hospital, School of Medicine, Zhejiang University, Yiwu, 322000, China; e Zhejiang Provincial Key Laboratory for Microbial Biochemistry and Metabolic Engineering, Hangzhou, 310058, China

**Keywords:** Genome mining, Microbial natural product, DNA intercalator, UvrA-like protein, Biosynthetic gene clusters, Tetracycline

## Abstract

Microbial natural products have been one of the most important sources for drug development. In the current postgenomic era, sequence-driven approaches for natural product discovery are becoming increasingly popular. Here, we develop an effective genome mining strategy for the targeted discovery of microbial metabolites with antitumor activities. Our method employs *uvrA*-like genes as genetic markers, which have been identified in the biosynthetic gene clusters (BGCs) of several chemotherapeutic drugs of microbial origin and confer self-resistance to the corresponding producers. Through systematic genomic analysis of gifted actinobacteria genera, identification of *uvrA*-like gene-containing BGCs, and targeted isolation of products from a BGC prioritized for metabolic analysis, we identified a new tetracycline-type DNA intercalator timmycins. Our results thus provide a new genome mining strategy for the efficient discovery of antitumor agents acting through DNA intercalation.

## 1 Introduction

Microbial specialized metabolites are rich sources of antibiotics and clinically used drugs [[1], [2], [3]]. These small molecules disrupt essential cellular processes through various modes of action. Historically, compounds of interest were isolated through large-scale screening of microbial culture extracts followed by activity tracking based on specific bioactivity assays. However, this traditional bioactivity-guided strategy is becoming less efficient due to the high rate of rediscovery of known compounds. In recent years, great efforts have been made to elucidate the biosynthetic pathways of microbial specialized metabolites with diversified structures and activities [4,5]. These studies not only revealed novel enzymology involved in the microbial specialized metabolism but also significantly increased our understanding of the self-resistance mechanisms of the producers, as it was found that self-resistance genes are frequently clustered with biosynthetic genes [6]. This co-localization property facilitates the identification of biosynthetic genes of a given microbial metabolite and offers genome mining approaches for the discovery of new compounds with predictable modes of action in the postgenomic era. Indeed, several self-resistance-guided strategies for identifying new bioactive compounds have been reported [[7], [8], [9], [10], [11], [12]].

**Fig. 1:**
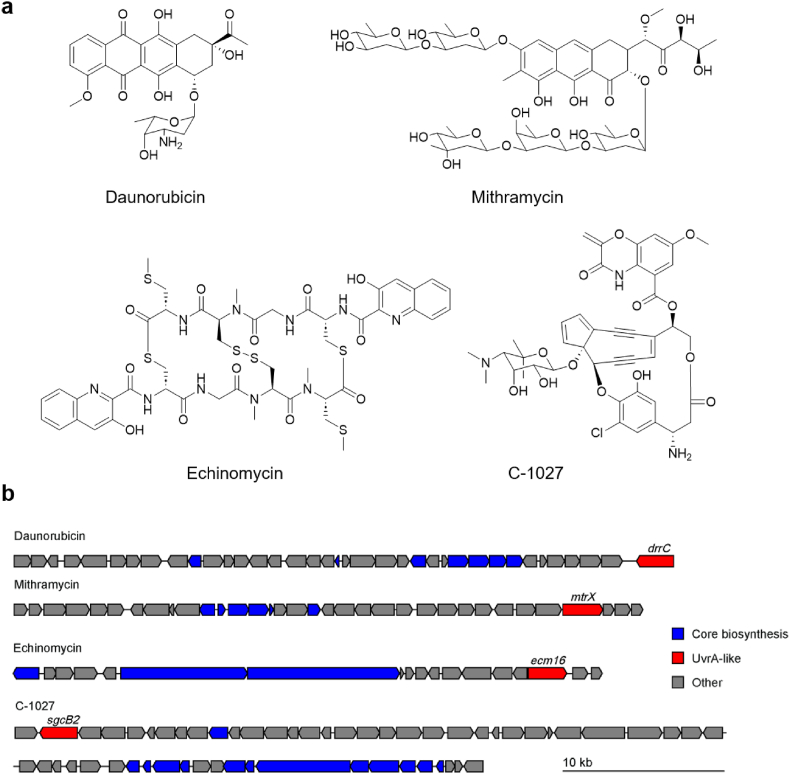
Selected antitumor microbial natural products that target DNA through noncovalent interactions. (a) Examples of antitumor microbial natural products. (b) The biosynthetic gene clusters (BGCs) of compounds in (a). The *uvrA*-like genes are highlighted in red.

One family of bacteria-derived antitumor and antimicrobial agents is DNA intercalators, which can insert into the DNA double helix through noncovalent interactions, leading to DNA structural changes and replication arrest [13,14]. Molecules from this class include chemotherapeutic anthracycline doxorubicin, enediyne C-1027, depsipeptide thiocoraline, and aurealic acid mithramycin [14]. These molecules are biosynthetically assembled by different enzymatic machinery and are structurally distinct from each other. It was found that the biosynthetic gene clusters (BGCs) of these compounds encode UvrA-like proteins that can confer self-protection to the producers. Examples include DrrC from the daunorubicin BGC [15], Ecm16 from the echinomycin BGC [16], and MtrX from the mithramycin BGC [17] (Fig. 1). Canonical UvrA is part of the UvrABCD nucleotide excision repair (NER) system [18]. UvrA participates in the initial ATP-dependent DNA scanning and damage recognition through direct UvrA-DNA interactions. This is followed by the recruitment of UvrB and UvrC for nucleotide excision. Although the detailed self-resistance mechanism of the UvrA-like protein family is still not fully understood, preliminary studies of DrrC and Ecm16 suggested that they also depend on ATP for their normal functions [19]. However, unlike canonical UvrA, they lack a UvrB-binding domain and can render self-protection independent of the host UvrABC system.

In this study, we explored the potential of utilizing the *uvrA*-like gene as a genetic marker for the targeted isolation of new anticancer and antimicrobial agents through genome mining. Through systematic genome analysis of gifted Actinobacteria genera, prediction and classification of UvrA-like gene-containing BGCs, and metabolic analysis of a strain prioritized for target compound isolation, we identified new tetracycline-type DNA intercalator timmycins. Together, our results reported a new genome mining strategy for the efficient isolation of antibacterial and antitumor agents acting through DNA intercalation.

## 2 Material and methods

### 2.1 General methods

DNA primers were purchased from Tsingke Biological Technology. Reagents were purchased from Sigma‒Aldrich, New England Bio Labs, and Bio Basic Inc. DNA manipulations of *Escherichia coli* and *Actinomadura* were carried out according to standard procedures. Strain *Actinomadura* sp. ATCC 31491 was maintained on MSF agar (2% (w/v) mannitol, 2% soy flour, 2% agar). Apramycin (50 μg mL^−1^), kanamycin (50 μg mL^−1^), and nalidixic acid (25 μg mL^−1^) were used for the selection of recombinant strains.

### 2.2 Gene inactivation in *Actinomadura* strain

For the construction of a gene knock-out mutant in *Actinomadura* sp. ATCC 31491, two ∼3 kb homologous arms flanking the targeted region were amplified by PCR using genomic DNA of *Actinomadura* sp. ATCC 31491 as templates. These segments were assembled into linearized pYD69 through seamless cloning [20]. After confirmation by DNA sequencing, the obtained vector was introduced into methylation-deficient *E. coli* ET12567/pUZ8002 for *E. coli*-*Actinomadura* conjugation. Exconjugants were obtained after selection for apramycin resistance. After several rounds of nonselective growth, replica plating and PCR were then used to screen the apramycin-sensitive colonies for gene knockout mutants (Fig. S1).

### 2.3 Metabolic analysis of *Actinomadura* strains

For the metabolic analysis, strain *Actinomadura* sp. ATCC 31491 and its mutant strain were grown on MSF plates at 30 °C for 10 days until sporulation. These MSF agar plates were then sliced into small pieces, extracted by ethyl acetate, and subjected to HPLC analysis. Analytic HPLC analysis was carried out with an Agilent 1260 HPLC apparatus using an Elipse EC-C18 column (5 μm, 4.6 mm ID × 150 mm) (detection wavelength: 280 nm). Elution was performed at 1.0 mL/min with a mobile-phase mixture consisting of a linear gradient of water and acetonitrile (both contain 0.05% (v/v) formic acid) as follows: 30%–90% acetonitrile in 18 min, 90%–100% acetonitrile in 2 min, and then maintained at 100% acetonitrile for 3 min.

### 2.4 Compound isolation and structural elucidation

For isolation of compounds, the ethyl acetate extract of *Actinomadura* sp. ATCC 31491 grown on MSF agar was fractionated on a Sephadex LH-20 column with MeOH elution. After analysis with HPLC, the fractions containing metabolites of interest were combined, and the target compounds were purified from these fractions by reversed-phase semipreparative HPLC (YMC-Triart C18, 5 μm, 10 mm ID × 250 mm) (UV detection at 280 nm). Compounds **1** (40 mg, t_R_ = 33.0 min) and **2** (30.1 mg, t_R_ = 45.0 min) were purified with 52% (acetonitrile+ 0.05% FA)/(H_2_O + 0.05% FA) at 4 mL/min. The ^1^H- and ^13^C and 2D NMR spectra were recorded on a Bruker AV-600 MHz spectrometer using CDCl_3_ as the solvent.

### 2.5 Antibacterial and antitumor activity assays

*S. aureus* subsp. *aureus* Rosenbach ATCC 29213, *E. faecalis* ATCC 19433, and *P*. *aeruginosa* ATCC 27853 were used for antibiotic assays. Briefly, microbial seed cultures were initiated by inoculating 5 mL LB and by growing these overnight at 37 °C and 220 rpm. Each culture was then diluted to an initial OD_600_ of 0.02 in a 100 μL volume per well in a 96-well plate, which gave an inoculum of approximately 5 × 10^5^ to 5 × 10^6^. The wells contained varying concentrations of the compounds tested: 0, 0.096 nM, 19.3 nM, 96 nM, 193 nM, 386 nM, 0.96 μM, 1.93 μM, 3.86 μM, 9.6 μM, 19.3 μM, and 38.6 μM final concentration. Assays were set up in triplicate. Plates were incubated at 37 °C for 16 h. The compounds used for antibiotic assays included mithramycin, timmycin A, and timmycin B.

For cytotoxicity assays, compounds were tested against cell lines (A375, HCT116, HeLa). Cells were seeded as aliquots into 96-well plates at a density of 5 × 10^3^ cells per well in DMEM (Dulbecco's Modified Eagle Medium) supplemented with 10% FBS, 50 IU/mL penicillin and 50 μg mL^−1^ streptomycin. After 24 h of incubation at 37 °C, compounds diluted in the same culture medium were added to the cells at final concentrations of 0.08 nM, 8 nM, 80 nM, 0.4 μM, 0.8 μM, 2 μM, 10 μM, and 50 μM. After an additional 48 h of incubation, the cells were first washed twice with PBS buffer, 120 μL of MTT (3-(4,5-dimethylthiazol-2-yl)-2,5-diphenyltetrazolium bromide) (0.83 mg/mL) solution was added to each well [21], and plates were returned to the incubator for 4 h. After removal of the solution from each well, 150 μL of DMSO was added to lyse the cells and solubilize the purple crystals. The samples were read on a microplate detector at 490 nm. The obtained results were processed using GraphPad Prism 7 software.

## 3 Results and discussion

### 3.1 Genome mining of BGCs with *uvrA*-like genes

To explore the distribution of UvrA-like proteins in actinobacteria that are known as gifted natural product producers, we first collected sequences of six UvrA-like proteins from BGCs of known antitumor compounds. These include DrrC (NCBI accession number: AAB39274), QncO (AGD95041), MtrX (CAK50795), SgcB2 (ANY94424), Ecm16 (BAE98165), and Luz28 (UKU0991). We then used these sequences as queries for the tblastn searches against the NCBI database (July 2022) of RefSeq genomes of selected *Actinobacteria* genera (*Actinomycetales*, *Frankiales*, *Micromonosporales*, *Streptomycetales*, and *Streptosporangiales*) in the GenBank database [22]. Homologous proteins with sequence identity ≥30% and alignment length >600 amino acids were collected. After dereplication, 3820 actinobacteria genomes containing approximately six thousand UvrA/UvrA-like proteins were recovered. Next, the sequences of 40 kb upstream and downstream regions of each *uvrA*/*uvrA*-like gene were collected and then subjected to antiSMASH 6.0 analysis to detect the potential presence of putative BGCs [23]. These efforts resulted in 642 BGCs in total.

**Fig. 2:**
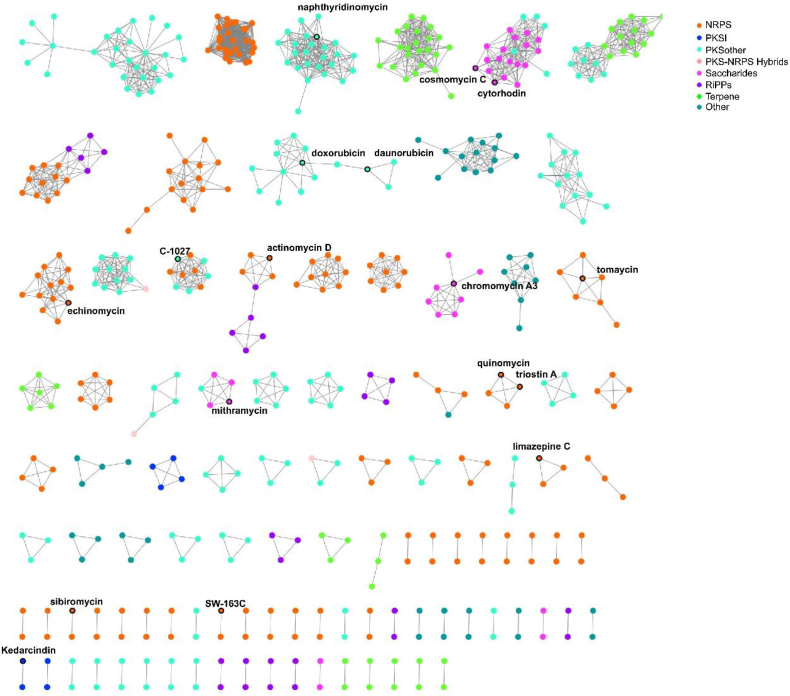
Sequence similarity network (SSN) of BGC obtained using BiG-SCAPE and visualized with Cytoscape 3.9.1. Each node represents a BGC. MiBIG BGCs are labeled with the corresponding product.

We then used the BiG-SCAPE tool to construct the sequence similarity networks of these BGCs and group them into eight classes, including PKSs, other PKSs, NRPSs, PKS-NRPS hybrids, RiPPs, saccharides, terpenes, and others [24] (Fig. 2). Our present study focused on the saccharide class for further study. All BGCs from this class contain type II PKS and glycosyltransferase genes, which potentially encode aromatic polyketides decorated with saccharide residues. BGCs of characterized compounds fall into this category, including those for mithramycin, chromomycin A3, cytorhodin Y, and cosmomycin C [[25], [26], [27], [28]]. Among them, mithramycin has been approved for clinical anticancer use [25]. Carbohydrate moieties have been shown to be essential for the biological properties of these molecules.

### 3.2 In silico analysis of the tim BGC that is prioritized for further study

**Fig. 3:**
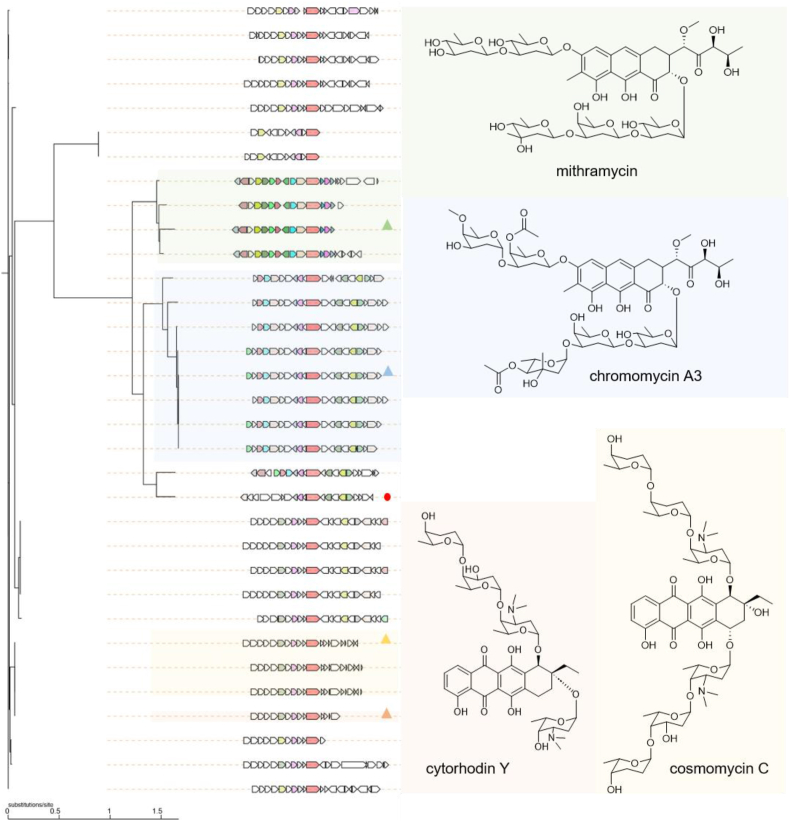
CORASON phylogenetic tree of the saccharide class BGCs. Representative BGCs from MiBIG are marked with triangles, and the corresponding natural products are also displayed. The BGC prioritized for further metabolite characterization is labeled with a red circle.

**Fig. 4:**
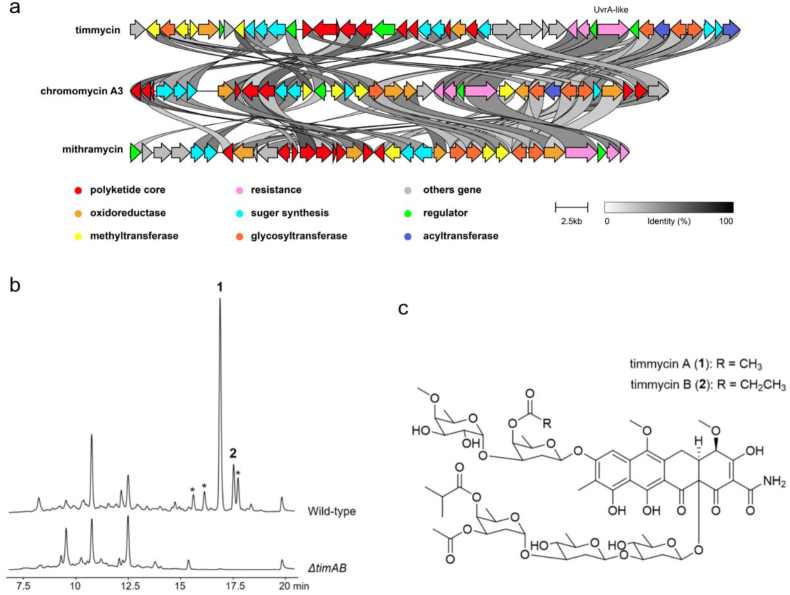
The biosynthetic gene cluster and structures of timmycin. (a) Comparison of the timmycin BGC with the mithramycin and chromomycin A3 BGCs. (b) Metabolic profiling of *Actinomadura* sp. ATCC 31491 and its Δ*timAB* mutant. (c) Structures of timmycin A and B.

Next, we used CORASON to construct a phylogenetic tree of these UvrA-like protein-containing BGCs from the saccharide class [24] (Fig. 3). One BGC from the strain *Actinomadura* sp. ATCC 31491 caught our attention and was prioritized for metabolite characterization (Fig. 3). This BGC (named here as *tim* BGC) is phylogenetically related to the BGCs of aureolic acids mithramycin and chromomycin, but it falls into an isolated clade. Comparison of the *tim* BGC to those of mithramycin (*mtm* BGC) and chromomycin (*cmm* BGC) revealed that the *tim* BGC lacks a pair of genes (*mtmOIV/mtmW*, *cmmOIV/cmmW*) that are conserved in the *mtm* and *cmm* BGCs (Fig. 4a, Table S4). These genes encode enzymes responsible for the oxidative cleavage of the fourth ring of a tetracyclic precursor, resulting in a tricyclic structure. In addition, the *tim* BGC encodes a putative amidotransferase (TimD), the homolog of which has been demonstrated to catalyze the transamination of malonate to malonamate in the biosynthesis of oxytetracycline, which introduces an amide unit at one terminus of the polyketide backbone [29]. The presence of this gene in *tim* BGC suggested that the corresponding product possesses a tetracycline-type amidated aglycon structure.

### 3.3 Identification and structure elucidation of timmycins

To identify the product arising from the *tim* BGC, we first generated a mutant strain of *Actinomadura* sp. ATCC 31491 by deleting the *timA* and *timB* genes, which encode the type II PKS ketoacyl synthase KS_α_ and KS_β_, respectively. We then compared the metabolic profiling of strain Δ*timAB* with that of the parental strain ATCC 31491 by HPLC analysis of their culture extracts. After screening different culture media, we identified a set of peaks that were only present in the culture extract of the parental strain but not in that of the strain Δ*timAB* (Fig. 4b). We speculated that these peaks, which displayed similar UV‒vis spectra, are structural analogs and could derive from the *tim* BGC.

To further structurally characterize these compounds, we set out to isolate the two major components (**1** and **2**) from the large-scale culture of strain *Actinomadura* sp. ATCC 31491. Compound **1** (named as timmycin A) displayed a mass value of *m*/*z* 1316.4943 from HR-ESI-MS, corresponding to the molecular formula C_61_H_83_NO_29_ (calcd for 1316.4943, [M+Na]^+^) (Fig. S2a). Compound **2** (named as timmycin B) had a molecular formula of C_62_H_85_NO_29_ (observed: 1330.5110, calcd for [M+Na]^+^: 1330.5099) (Fig. S2b). Their structures were elucidated using a combination of 1D and 2D NMR analysis (Fig. 4c and Fig. S3–6). Timmycins contain a tetracycline-type aglycone substituted with two oligosaccharide side chains, including a disaccharide attached to C-8 and a trisaccharide attached to C-12a of the aglycone (Fig. 4c). The trisaccharide chain consists of d-olivose (sugar C), d-olivose (sugar D), and 4-*O*-isobutyryl-3-*O*-acetyl-d-olivose (sugar E), while the disaccharide contains 4-*O*-methyl-d-fuctose (sugar B) and 4-*O*-acetyl-d-oliose (sugar A) for timmycin A or 4-*O*-propionyl-d-oliose for timmycin B (Fig. S7). Timmycins are new glycosylated aromatic polyketides and are distinct from mithramycin and chromomycin in both aglycone and saccharide patterns.

### 3.4 Proposed biosynthetic pathway of timmycins

**Fig. 5:**
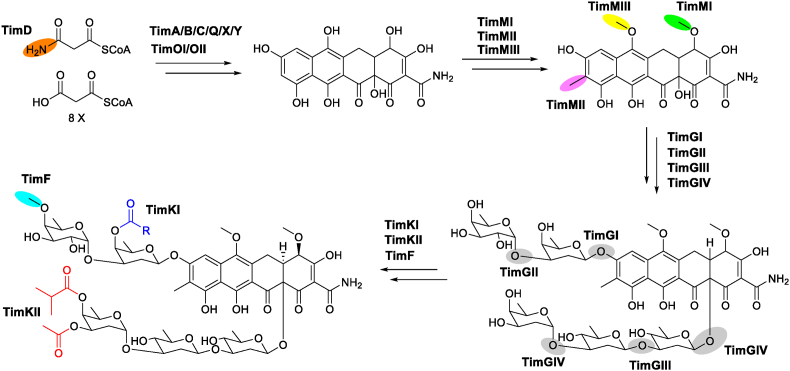
The proposed biosynthetic pathway of timmycins.

Based on the established knowledge of the biosynthetic pathways of mithramycin/chromomycin and other aromatic polyketides [25,26], we proposed a biosynthetic route to timmycins (Fig. 5). The *tim* BGC encodes enzymes necessary for the construction of an aminated tetracyclic core. These include KS_α_ (TimA), KS_β_ (TimB), acyl carrier protein (TimC), amidotransferase (TimD), aromatase (TimQ), and cyclases (TimY and TimX). Oxygenases TimOI and TimOII are suggested to introduce hydroxyl groups into the polyketide core. The putative C-methyltransferase TimMII and the two O-methyltransferases TimMI and TimMIII could be responsible for three methylation steps occurring on the timmycin aglycone. For the subsequent glycosylation steps, the four glycosyltransferases (TimGI/GII/GIII/GIV) are expected to catalyze the formation of five glycosidic bonds, which are analogous to those of the mithramycin and chromomycin pathways. More specifically, TimGIV and TimGIII, which are similar to CmmGIV (57% identity) and CmmGIII (53% identity) from the chromomycin pathway (Table S4), are assumed to sequentially transfer sugar C (by TimGIV), sugar D (by TimGIII), and sugar E (by TimGIV) to the aglycon, whereas TimGI and TimGII, which are more closely related to CmmGI (53% identity) and CmmGII (52% identity), are suggested to install sugars A and B, respectively. As the last steps in timmycin biosynthesis, the sugar *O*-methyltransferase TimF and the two acyltransferases TimKI and TimKII could further tailor the sugar moieties through methylation and acylations.

### 3.5 Biological activities of timmycins

We next tested the antibacterial and cytotoxic activity of timmycins (Table 1, Table 2). Both **1** and **2** have strong antibacterial activity against Gram-positive bacteria, including *Staphylococcus aureus* subsp. *aureus* ATCC 29213 and *Enterococcus faecalis* ATCC 19433 (Table 1). No antifungal or anti-Gram-negative bacterial activity was observed at the concentration we tested. Compound **1** displays cytotoxicity against cancer cell lines (A375, HCT116, and HeLa) in the submicromolar range (Table 2).

**Table 1:** Minimum inhibitory concentration (MIC) values of timmycin A and B (**1** and **2**) against common pathogens.

Assays	MIC/μM		
	Mithramycin	Timmcycin A (1)	Timmcycin B (2)
*Staphylococcus aureus* subsp. *aureus* ATCC 25923	3.6	0.7	0.6
*Enterococcus faecalis* ATCC 19433	4.7	3.1	3.4
*Pseudomonas aeruginosa* ATCC 27853	>38.6	>38.6	>38.6
*Escherichia coli* ATCC 25922	–	>38.6	>38.6
*Stenotrophomonas maltophilia* ATCC 13637	–	>38.6	>38.6
*Klebsiella pneumoniae* subsp. *pneumoniae* ATCC 13883	–	>38.6	>38.6
*Candida albicans* ATCC 90028	–	>38.6	>38.6

**Table 2:** Cytotoxic assays against selected cancer cell lines.

Assays	IC_50_/μM		
	A375	HCT116	HeLa
Mithramycin	0.018	0.015	0.032
Doxorubicin	0.015	0.065	0.166
Timmcycin A (**1**)	0.228	0.194	0.195

To determine the ability of **1** to interact with DNA, we used a gel mobility retardation assay by incubation of **1** with a 0.7 kb genomic DNA fragment randomly selected from the genome of *Actinomadura* sp. ATCC 31491 (Fig. S8). We found that **1** affects the mobility of the DNA fragment, indicating that **1** can bind DNA. Next, we investigated the role of the UvrA-like protein TmrX in self-resistance. We introduced *tmrX* into the heterologous host *Streptomyces albus* J1074. A disk diffusion test was then used to evaluate the sensitivity of the resulting strain *S. albus* + *tmrX* to **1** (Fig. S9). Compared with the control strain *S. albus,* the resistance of *S. albus* + *tmrX* to **1** increased significantly. Together, these results supported that **1** exerts its antibacterial and cytotoxic action by interacting with DNA.

## 4 Conclusion

In this study, we developed a new genome mining strategy for the targeted discovery of bacteria-derived antitumor agents. This method is based on the previous observation that the biosynthetic gene clusters of natural DNA intercalators encode a family of UvrA-like proteins for self-resistance, indicating the potential of the *uvrA*-like gene as a genetic marker to guide the identification of BGCs for new DNA intercalators. As a proof of concept, we successfully isolated a new tetracycline-type DNA intercalator, timmycin, from an *Actinomadura* strain. Although self-resistance gene-guided genome mining approaches have been described previously, this is the first time that the *uvrA*-like gene family is used for the targeted isolation of microbial bioactive metabolites. Considering that hundreds of new *uvrA*-like gene-containing BGCs were identified in this study, our genome mining strategy has great potential for the discovery of novel anticancer drug candidates.

## Declaration of competing interest

The authors declare that they have no conflicts of interest.

## CRediT authorship contribution statement

**Zhijie Zhao:** Data curation, Formal analysis, Investigation, Visualization, Writing – original draft. **Guiyun Zhao:** Data curation, Formal analysis, Investigation, Visualization. **Yi Chai:** Investigation, Visualization. **Wei Li:** Investigation. **Kaihui Song:** Investigation. **Wenbin Zhao:** Investigation. **Hairong Li:** Investigation. **Miaolian Wu:** Writing – review & editing. **Zhan Zhou:** Conceptualization, Methodology, Project administration, Resources, Supervision, Validation, Writing – original draft, Writing – review & editing. **Yi-Ling Du:** Conceptualization, Funding acquisition, Methodology, Project administration, Resources, Supervision, Validation, Writing – original draft, Writing – review & editing.
