# Supplementary material for: Targeted genome mining for microbial antitumor agents acting through DNA intercalation
Source: Synth Syst Biotechnol. 2023 Jul 22;8(3):520–6. doi: 10.1016/j.synbio.2023.07.003 (PMC10413001; doi:10.1016/j.synbio.2023.07.003)
Supplement: Multimedia component 1 [file mmc1.docx]

**Supporting Information**

**Targeted genome mining for microbial antitumor agents acting through DNA intercalation**

Zhijie Zhao^a^, Guiyun Zhao^a^, Yi Chai^b^, Wei Li^a^, Kaihui Song^a^, Wenbin Zhao^c^, Hairong Li^c^, Miaolian Wu^d^, Zhan Zhou^c,d^*, Yi-Ling Du^a,d,e^*

^a^The Fourth Affiliated Hospital and Department of Microbiology, School of Medicine, Zhejiang University, Hangzhou, 310058, China;

^b^Yong Loo Lin School of Medicine, National University of Singapore, Singapore;

^c^Innovation Institute for Artificial Intelligence in Medicine, College of Pharmaceutical Sciences, Zhejiang University, Hangzhou, 310058, China;

^d^The Fourth Affiliated Hospital, School of Medicine, Zhejiang University, Yiwu, 322000, China;

^e^Zhejiang Provincial Key Laboratory for Microbial Biochemistry and Metabolic Engineering, Hangzhou 310058, China

# **Table S1.** Plasmids and strains in this study.

| Plasmids/strains | Description | Sources |
| --- | --- | --- |
| pYD69 | Used for gene inactivation | laboratory stock |
| pSOK804KT | Used for intergration *tmrX* into host with *kasOp** | laboratory stock |
| *Actinomadura* sp. ATCC  31491 | Wild-type timmycin producer | laboratory stock |
| Δ*timAB* | ATCC 31491(Δ*timAB::*scar) | this study |
| *Streptomyces albus* J1074 | Heterologous host |  |
| J1074+*tmrX* | J1074+*tmrX* (under *kasOp**) | this study |
| *S. aureus* ATCC 29213 | Antibacterial assays | this study |
| *E. faecalis* ATCC 19433 | Antibacterial assays | this study |
| *E. coli* ATCC 25922 | Antibacterial assays | this study |
| *P*. *aeruginosa* ATCC 27853 | Antibacterial assays | this study |
| *Stenotrophomonas maltophilia* ATCC 13637 | Antibacterial assays | this study |
| *Klebsiella pneumoniae* subsp. *pneumoniae* ATCC 13883 | Antibacterial assays | this study |
| *Candida albicans* (Robin) Berkhout ATCC 90028 | Antibacterial assays | this study |
| *E.coli* DH5α | General cloning host | laboratory stock |
| *E. coli* ET12567/pUZ8002 | Methylation-deficient strains used for conjugation | laboratory stock |
| A375 | Cytotoxic assays | this study |
| HCT116 | Cytotoxic assays | this study |
| HeLa | Cytotoxic assays | this study |

#

# **Table S2**. Primers in this study.

| Primers | Sequence (5’→3’) | Description |
| --- | --- | --- |
| chr-U-NcoI-F2 | cctagggcgtgcccatggccaaggccgcgatggagagcctg | Primers for deletion *timAB* |
| chr-U-R2 | tcgatcaggtcgccgtgggggcacagccggatctccgacggtc |  |
| chr-D-F | ccacggcgacctgatcgacctgg |  |
| chr-D-NcoI-R2 | ttataaaagcttccatggcgccggagtgaggagcagcgcac |  |
| 152linear-F | gaattcgtaatcatgtcatagctgtttc | Primers for amplification of linear pYD69 |
| 152linear-R | aagcttgttgatgacgacattgaccattc |  |
| chr-F | cgtcctggtgcagcgtgaacgc | Primers for screening the double-crossover mutants of Δ*timAB* |
| chr-R | gccagcgagtcgtagcccaggg |  |
| 3175-NdeI-F | gtgccgcgcggcagccatatgaccgacagcaccggcggcg | Primers for amplification *tmrX* |
| 3175-HindⅢ-R | ctcgagtgcggccgcaagcttgcccggtccgcgttcagctcttgag |  |
| CS1 | gtgttccccgcgtcacgcagctc | Primers for screening the mutants of *tmrX* |
| luz7-Nde1-F2 | AGCAGCCATATGgtggtcgcgaaaccctcaactcccgc | Primers for amplifying GC rich fragment |
| luz7-Xba1-R2 | AGCAGCTCTAGAgctcatgtgtcctgcaacaggcgc |  |

| Protein | Size^a^ | Proposed function | Similar protein | S/I (%) | Accession number^b^ |
| --- | --- | --- | --- | --- | --- |
| TimV | 390 | β-lactamase family protein |  | 100/71.8 | NMH82297.1 |
| TimMII | 345 | C-methyltransferase | MmyMII | 94/52.2 | QVQ68782.1 |
| TimGI | 390 | Glycosyltransferase | MmyGI | 97/35.9 | QVQ68810.1 |
| TimMI | 340 | *O*-methyltransferase | MmyMI | 98/61.1 | QVQ68781.1 |
| TimMIII | 219 | Methyltransferase | NocQ | 62/40.8 | ADR01068.1 |
| TimOII | 530 | Oxygenase | MmcT | 95/39.3 | AAD32744.1 |
| TimRII | 119 | BlaI/CopY family transcriptional regulator |  | 93/60.3 | NUR02277.1 |
| TimH | 271 | M56 family metallopeptidase |  | 87/45.7 | WP_204052558.1 |
| TimF | 257 | Sugar *O*-methyltransferase | MycF | 81/60.8 | BAA03673.1 |
| TimUII | 262 | 4-ketoreductase | ChlC5 | 93/45.9 | AAZ77680.1 |
| TimUI | 326 | 3-ketoreductase | ChlC4/MmyU | 98/48.9 | AAZ77681.1 |
| TimSIV | 480 | Hexose 2, 3-dehydratase | Sim20 | 97/47.1 | AAL15606.1 |
| TimRI | 276 | Transcriptional regulator | MmyRI | 92/47.0 | QVQ68801.1 |
| TimTII | 251 | Ketoreductase | MmyTII | 100/62.8 | QVQ68783.1 |
| TimD | 616 | Amidotransferase | SsfD | 100/70.9 | ADE34521.1 |
| TimC | 82 | ACP | SsfC | 95/58.9 | ADE34520.1 |
| TimB | 410 | Chain length factor (KS*_β_*) | SsfB | 97/70 | ADE34519.1 |
| TimA | 426 | Ketosynthase (KS*_α_*) | SsfA | 98/77.8 | ADE34518.1 |
| TimRIV | 564 | TetR family transcriptional  regulator |  | 51/37.2 | AGL15462.1 |
| TimY | 256 | Cyclase | MmyY | 100/66.5 | QVQ68794.1 |
| TimTI | 255 | Ketoreductase | MmyTI | 99/53.7 | QVQ68796.1 |
| TimSII | 338 | Hexose-4-ketoreductase | PokS6 | 89/46.3 | ACN64824.1 |
| TimEI | 355 | NDP-glucose synthetase | Lct49 | 100/59.4 | ABX71132.1 |
| TimX | 147 | Cyclase | MmyX | 93/56.5 | QVQ68793.1 |
| TimOI | 398 | Oxygenase | MmyOI | 98/49.8 | QVQ68795.1 |
| TimQ | 299 | Aromatase | MmyQ | 95/46.7 | QVQ68785.1 |
| TimEII | 327 | NDP-hexose-4,6-dehydratase | Lct48 | 96/74.4 | ABX71131.1 |
| TimL | 637 | Acyl-CoA ligase | MmyL | 78/45.1 | QVQ68786.1 |
| TimPII | 572 | Carboxyl transferase | DacP3 | 93/60.4 | AFU65907.1 |
| TimJ | 169 | Biotin carboxylase carrier protein | TamJ | 81/50.3 | AFY23041.1 |
| TimPI | 458 | Biotin carboxylase | DacP1 | 95/68.3 | AFU65905.1 |
| TmrB | 252 | Membrane protein | MmrBII | 100/53.7 | QVQ68817.1 |
| TmrA | 318 | ATP binding protein | MtrA | 96/57.6 | CAK50797.1 |
| TimRV | 185 | PadR family transcriptional  regulator |  | 99/54.3 | AGG12549.1 |
| TmrX | 815 | UV-repair system | MmrX | 99/54.4 | QVQ68812.1 |
| TimRIII | 240 | Transcriptional regulator | MmyRIII | 90/38.1 | QVQ68818.1 |
| TimGIII | 386 | Glycosyltransferase | MmyGIII | 99/49.1 | QVQ68808.1 |

# **Table S3** The annotation of *tim* BGC.

| TimKI | 410 | *O*-acyltransferase | TxnB11 | 91/33.7 | AKT74303.1 |
| --- | --- | --- | --- | --- | --- |
| TimGIV | 408 | Glycosyltransferase | MmyGIV | 99/46.0 | QVQ68807.1 |
| TimGII | 380 | Glycosyltransferase | MmyGII | 96/45.1 | QVQ68809.1 |
| TimSI | 288 | dTDP-4-dehydrorhamnose 3, 5-epimerase | CalS2 | 87/50.5 | AAM94769.1 |
| TimSIII | 203 | dNDP-hexose 3, 5-epimerase | PokS7 | 92/45.4 | ACN64855.1 |
| TimKII | 437 | Acyltransferase | TxnB11 | 86/37.5 | AKT74303.1 |

1. Amino acid number
2. NCBI accession number

# **Table S4.** Gene annotations of the timmycin BGC and its comparison with the BGCs of mithramycin (*mtm*) and chromomycin A3 (*cmm*).

| Timmycin BGC | Chromomycin A3 BGC | Mithramycin BGC | Proposed function | % aa identity (Tim: Cmm/Mtm) |
| --- | --- | --- | --- | --- |
| TimKI | CmmA |  | *O*-acyltransferase | 50.5/ |
| TimKII |  |  | acyltransferase |  |
|  |  | MtmA | AdoMet synthetase |  |
|  | CmmC | MtmC | C-methyltransferase |  |
| TimEI | CmmD | MtmD | NDP-glucose synthase | 56.4/54.8 |
| TimEII | CmmE | MtmE | NDP-4,6-dehydratase | 65.5/64.8 |
|  | CmmF |  | NDP-5-epimerase |  |
| TimGI | CmmGI | MtmGI | Glycosyltransferase | 52.9/42.3 |
| TimGII | CmmGII | MtmGII | Glycosyltransferase | 51.8/43.9 |
| TimGIII | CmmGIII | MtmGIII | Glycosyltransferase | 52/49.4 |
| TimGIV | CmmGIV | MtmGIV | Glycosyltransferase | 56.5/49.4 |
|  |  | MtmH | Adenosylhomocysteinase |  |
|  |  |  | Hydrolase |  |
| TimB | CmmK | MtmK | Ketosynthase (KS_β_) | 65/67.8 |
|  | CmmLI |  | Acyl-CoA ligase |  |
| TimL | CmmLII | MtmL | Acyl-CoA ligase | 47.2/45.6 |
| TimMI | CmmMI | MtmMI | O-methyltransferase | 53.4/58 |
| TimMII | CmmMII | MtmMII | C-methyltransferase | 59.6/52.6 |
| TimMIII |  |  | Methyltransferase |  |
| TimF | CmmMIII |  | *O*-methyltransferase | 70.6/ |
| TimOI | CmmOI | MtmOI | Oxygenase | 54.2/54 |
| TimOII | CmmOII | MtmOII | Oxygenase | 33.3/32.4 |
|  |  | MtmOIII | Oxygenase |  |
|  |  |  | Oxygenase |  |
|  | CmmOIV | MtmOIV | Oxygenase |  |
| TimA | CmmP | MtmP | Ketosynthase (KSα) | 68/67.4 |
| TimQ | CmmQ | MtmQ | Aromatase | 46.4/47.5 |
| TimRI | CmmRI | MtmR | Transcriptional activator | 52.3/40.2 |
| TimRIII | CmmRII | MtmY | Regulator | 56.1/33.3 |
| TimRII |  |  | Regulator |  |
| TimRIV |  |  | Regulator |  |
| TimRV |  |  | Regulator |  |
|  | CmmS | MtmS | Acyl carrier protein |  |
| TimC |  |  | Acyl carrier protein |  |
| TimTI | CmmTI | MtmTI | Ketoreductase | 51/52.8 |
| TimTII | CmmTII | MtmTII | Ketoreductase | 62/62.2 |
|  |  | MtmTIII | NDP-4-ketoreductase |  |
|  | CmmUI |  | NDP-5-ketoreductase |  |
|  | CmmUII |  | NDP-6-ketoreductase |  |
|  | CmmUIII |  | NDP-7-ketoreductase |  |
|  | CmmV | MtmV | NDP-2,3-dehydratase |  |
| TimUI | CmmW | MtmU | NDP-3-ketoreductase | 48/46.4 |
| TimUII |  |  |  |  |
|  | CmmWI | MtmW | Ketoreductase |  |
| TimX | CmmX | MtmX | Cyclase | 58/50.4 |
| TimY | CmmY | MtmY | Cyclase | 65/65.9 |
|  |  | MtmZ | Thioesterase |  |
| TmrA | CmrA | MtrA | ATP-binding protein | 59.9/57.7 |
|  |  |  | ATP-binding protein |  |
| TmrB | CmrB | MtrB | Membrane protein | 60/43.2 |
|  |  |  | Membrane protein |  |
| TmrX | CmrX | MtrX | UV-repair system | 59.5/53 |
| TimD |  |  | Amidotransferase |  |
| TimSI |  |  | dTDP-4-dehydrorham  -nose 3, 5-epimerase |  |
| TimSII |  |  | Hexose-4-ketoreductase |  |
| TimSIII |  |  | dNDP-hexose 3, 5-  epimerase |  |
| TimSIV |  |  | Hexose 2, 3-dehydratase |  |
| TimPI |  |  | Biotin carboxylase |  |
| TimJ |  |  | Biotin carboxylase carrier protein |  |
| TimPII |  |  | Carboxyl transferase |  |
| TimH |  |  | M56 family meta  -llopeptidase |  |
| TimV |  |  | serine hydrolase domain-containing protein |  |

#

**Figure S1.**

**
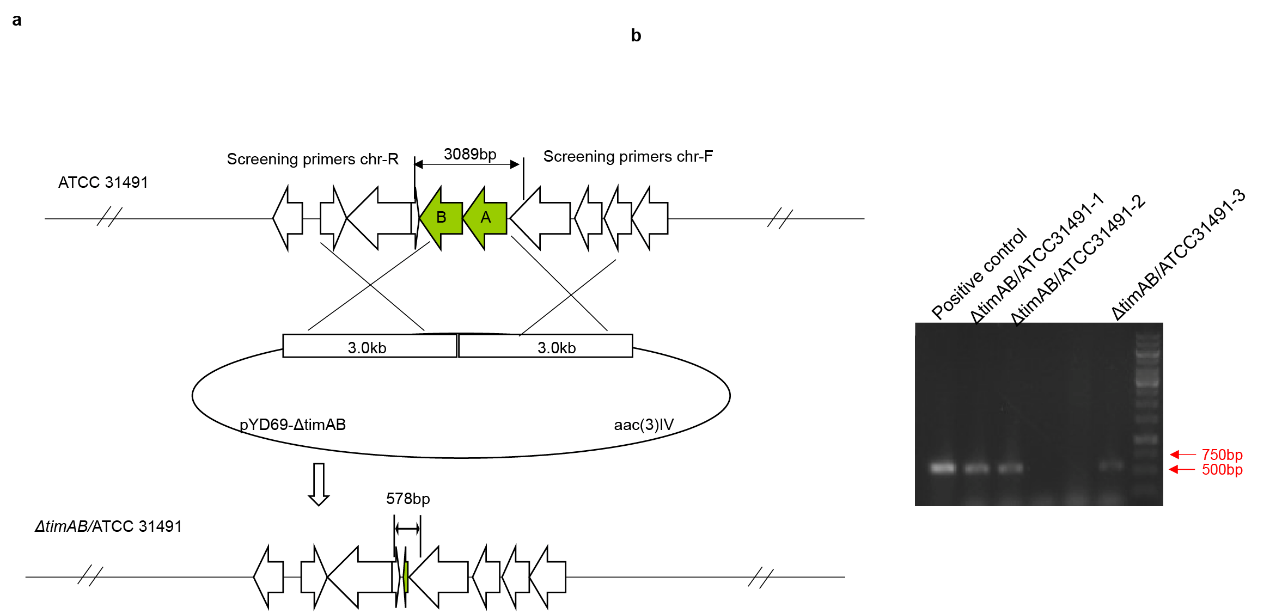
**

# Figure S1. Inactivation of genes *timAB* in *Actinomadura* sp. ATCC 31491. Left panel, genes inactivation of *timAB* by homologous recombination. Right panel, Screening of genes knockout mutants by PCR.

**Figure S2**

**(a)**


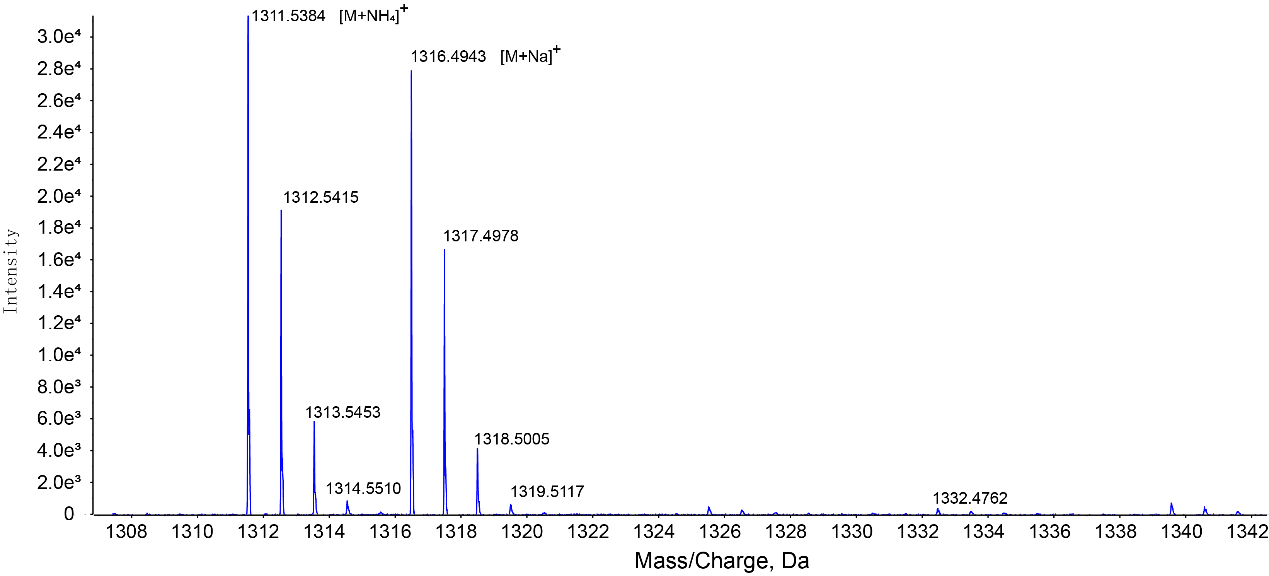


**(b)**

_
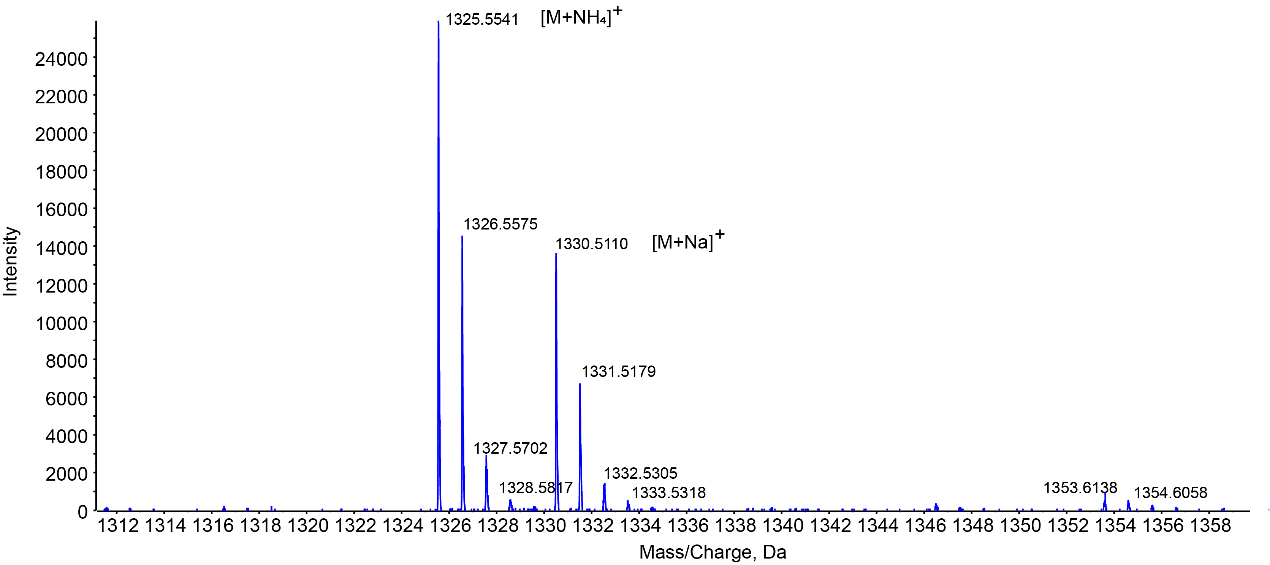
_

## **Figure S2**. HR-ESI-MS spectra of timmycin A (**a**) and B (**b**).

# **Figure S3.**

| Position | Timmycin A (**1**) | | Timmycin B (**2**) | |
| --- | --- | --- | --- | --- |
|  | ^1^H-NMR | ^13^C-NMR | ^1^H-NMR | ^13^C-NMR |
| 1 | - | 188.15 | - | 188.15 |
| 2 | - | 100.91 | - | 101.06 |
| 3 | - | 195.15 | - | 195.17 |
| 4 | 4.72 (1H, m) | 76.10 | 4.72 (1H, m) | 76.14 |
| 4a | 3.10 (1H, s) | 41.15 | 3.10 (1H, m) | 41.7 |
| 5 | 3.57 (1H, brs)  2.61 (1H, brs) | 19.96 | 3.57 (1H, brs)  2.63 (1H, m) | 20.02 |
| 5a | - | 123.08 | - | 123.09 |
| 6 | - | 143.64 | - | 143.63 |
| 6a | - | 133.20 | - | 133.37 |
| 7 | 6.97 (1H, s) | 95.79 | 6.97 (1H, s) | 95.91 |
| 8 | - | 159.99 | - | 159.97 |
| 9 | - | 112.21 | - | 112.15 |
| 10 | - | 156.99 | - | 156.83 |
| 10a | - | 109.01 | - | 108.78 |
| 11 | - | 162.32 | - | 162.52 |
| 11a | - | 107.70 | - | 107.65 |
| 12 | - | 199.45 | - | 199.45 |
| 12a | - | 81.61 | - | 81.91 |
| 13 | - | 173.52 | - | 173.59 |
| 3-OH | 15.22 (1H, s) |  | 15.68 (1H, s) |  |
| 10-OH | 8.99 (1H, s) |  | 9.13 (1H, s) |  |
| 11-OH | 9.94 (1H, s) |  | 9.92 (1H, s) |  |
| 13-NH_2_ | 9.14 (2H, s) |  | - |  |
| 14-OCH_3_ | 3.72 (3H, s) | 59.97 | 3.73 (3H, s) | 60.08 |
| 15-OCH_3_ | 3.67 (3H, s) | 60.14 | 3.68 (3H, s) | 60.31 |
| 16-CH_3_ | 2.17 (3H, s) | 8.47 | 2.17 (3H, s) | 8.32 |

| Sugar A |  |  |  |  |
| --- | --- | --- | --- | --- |
| A1 | 5.27 (1H, d, *J* = 9.9 Hz) | 96.95 | 5.27 (1H, m) | 97.30 |
| A2 | 2.30 (1H, m);2.16(1H, brs) | 31.10 | 2.30 (1H, m);2.16 (1H, m) | 31.31 |
| A3 | 4.03 (1H, m) | 70.43 | 4.03 (1H, m) | 70.61 |
| A4 | 5.15 (1H, m) | 69.59 | 5.17 (1H, brs) | 68.91 |
| A5 | 3.92 (1H, m) | 70.18 | 3.91 (1H, m) | 70.36 |
| A6 | 1.25 (3H, m) | 16.65 | 1.25 (3H, m) | 16.81 |
| A7-CO^－^ | - | 170.83 | - | 174.20 |
| A8-CH_3_ | 2.17 (3H, brs) | 20.67 | - | - |
| **2**-A9-CH_3_ | - | - | 1.18 (3H, t, *J* = 7.6 Hz) | 9.54 |
| **2**-A8-CH_2_^－^ |  |  | 2.44 (2H, q, *J* = 7.5 Hz) | 27.61 |
| Sugar B |  |  |  |  |
| B1 | 4.89 (1H, brs) | 96.96 | 4.91 (1H, brs) | 96.96 |
| B2 | 3.88 (1H, m) | 71.22 | 3.88 (1H, m) | 71.33 |
| B3 | 3.78 (1H, m) | 70.44 | 3.79 (1H, m) | 70.46 |
| B4 | 3.10 (1H, m) | 82.67 | 3.10 (1H, m) | 82.79 |
| B5 | 3.71 (1H, brs) | 68.76 | 3.71 (1H, brs) | 68.13 |
| B6 | 1.34 (3H, brs) | 17.78 | 1.32 (3H, brs) | 18.10 |
| B7-OCH_3_ | 3.52 (3H, s) | 60.17 | 3.53 (3H, s) | 60.43 |
| Sugar C |  |  |  |  |
| C1 | 5.41 (1H, d, *J* = 9.3 Hz) | 96.95 | 5.41 (1H, d, *J* = 9.7 Hz) | 97.02 |
| C2 | 2.37 (1H, m);1.69(1H, m) | 37.01 | 2.40 (1H, m);1.70 (1H, m) | 37.13 |
| C3 | 3.73 (1H, brs) | 81.86 | 3.64 (1H, brs) | 82.31 |
| C4 | 3.03 (1H, brs) | 74.66 | 3.06 (1H, brs) | 71.93 |
| C5 | 3.03 (1H, brs) | 71.59 | 3.06 (1H, brs) | 73.91 |
| C6 | 1.13 (3H, m) | 18.91 | 1.12 (3H, m) | 17.36 |
| Sugar D |  |  |  |  |
| D1 | 4.50 (1H, brs) | 99.95 | 4.50 (1H, brs) | 100.12 |
| D2 | 1.80 (1H, m); 1.70 (1H, m) | 31.01 | 1.97 (1H, m);1.70 (1H, m) | 30.06 |
| D3 | 3.63 (1H, m) | 69.06 | 3.70 (1H, m) | 74.10 |
| D4 | 3.52 (1H, m) | 70.88 | 3.62 (1H, m) | 69.26 |
| D5 | 3.48 (1H, m) | 68.93 | 3.53 (1H, m) | 69.16 |
| D6 | 1.34 (3H, m) | 17.79 | 1.34 (3H, m) | 16.80 |
| Sugar E |  |  |  |  |
| E1 | 4.93 (1H, brs) | 95.53 | 4.98 (1H, brs) | 95.25 |
| E2 | 2.16 (1H, brs); 1.89 (1H, m) | 35.34 | 2.19 (1H, brs); 1.84 (1H, m) | 35.35 |
| E3 | 5.24 (1H, m) | 68.64 | 5.24 (1H, m) | 68.73 |
| E4 | 4.71 (1H, m) | 74.06 | 4.72 (1H, m) | 73.89 |
| E5 | 3.94 (1H, m) | 66.29 | 3.91 (1H, m) | 66.46 |
| E6 | 1.10 (3H, m) | 17.17 | 1.14 (3H, m) | 16.80 |
| E7-C=O^－^ | - | 170.47 | - | 170.49 |
| E8-CH_3_ | 1.96 (3H, s) | 20.88 | 1.98 (3H, s) | 21.43 |
| E9-C=O^－^ | - | 176.45 | - | 176.41 |
| E10-CH^－^ | 2.50 (1H, m) | 33.91 | 2.51 (1H, m) | 34.16 |
| E11-CH_3_ | 1.10 (3H, m) | 18.91 | 1.12 (3H, m) | 19.04 |
| E12-CH_3_ | 1.10 (3H, m) | 19.01 | 1.12 (3H, m) | 18.82 |

# Figure S3. ^1^H (600 MHz) and ^13^C (150 MHz) NMR data of compounds **1** and **2** (CDCl_3_).

**Figure S4**

a. b.


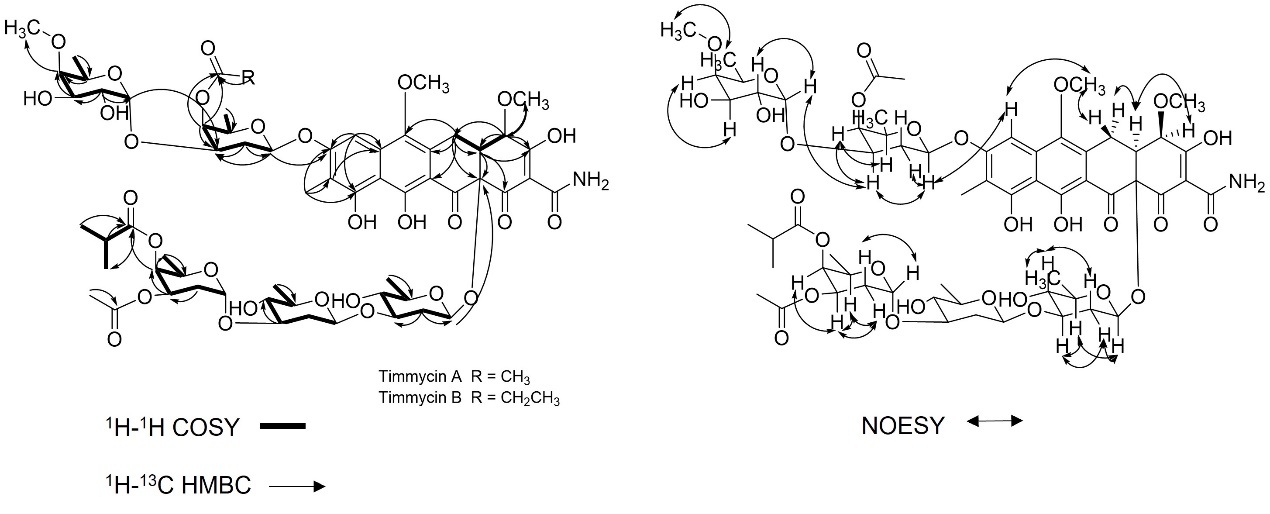


# Figure S4. 2D NMR correlations of timmycin A (**1**) and B (**2**). (a) Selected HMBC (→) and COSY (—) correlations of timmycin A and B. (b) Selected ROESY (↔) correlations of timmycin A.

**Figure S5**

**(a)**


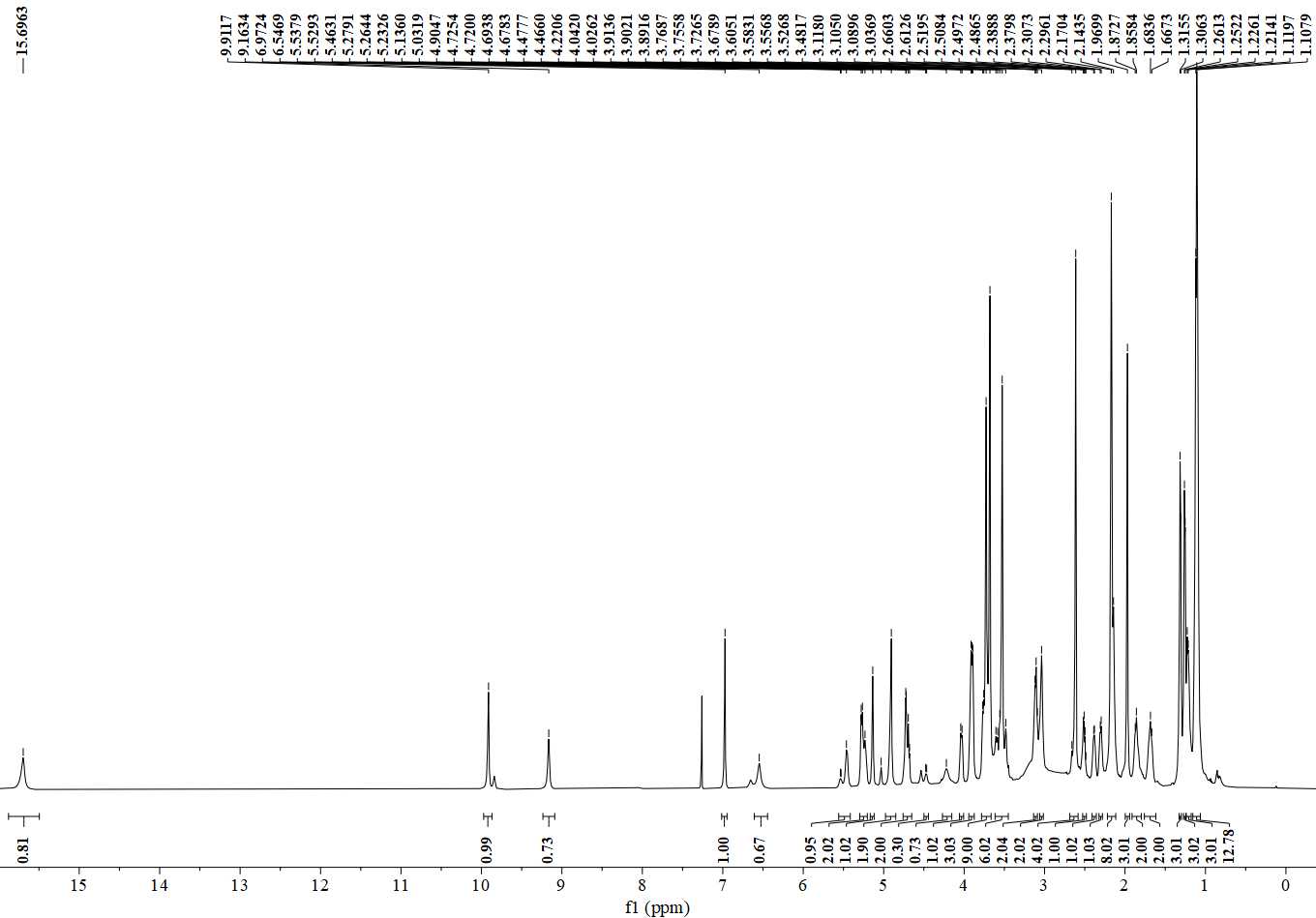


#

# (b)

#
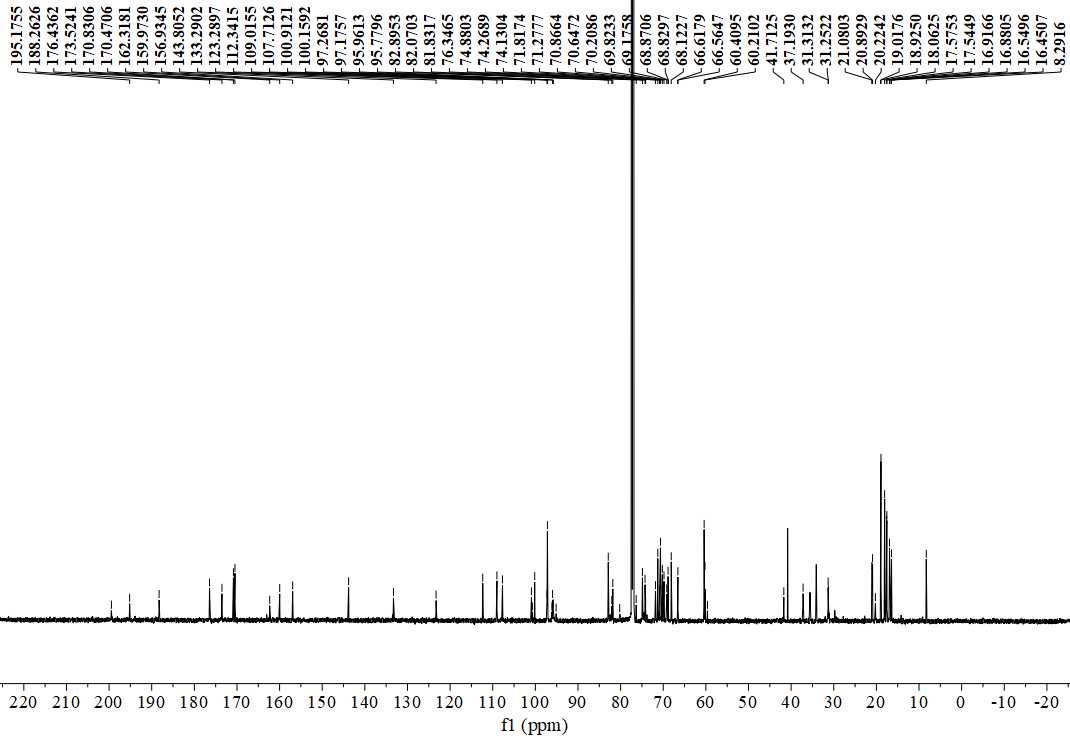


# (c)


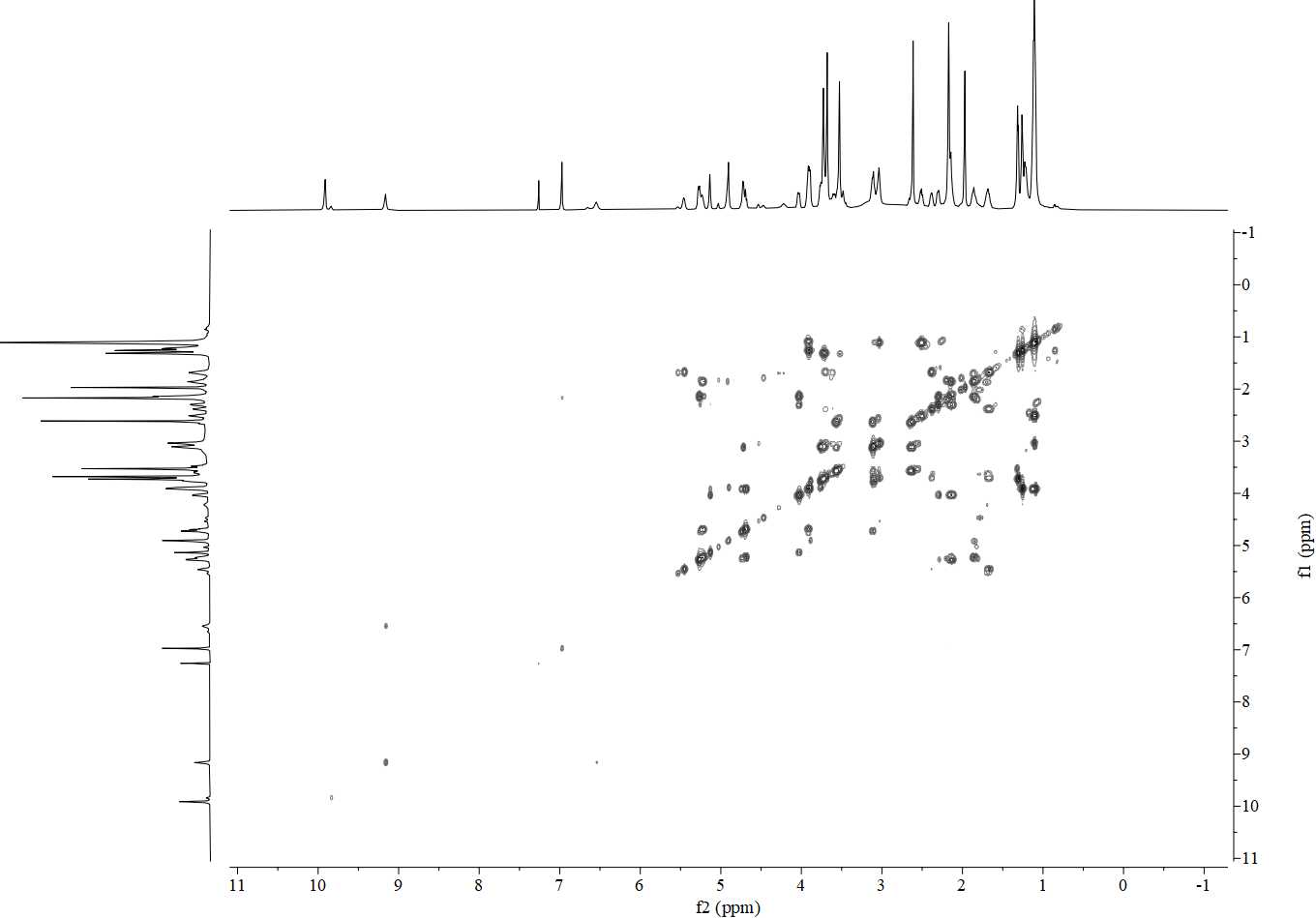


# (d)


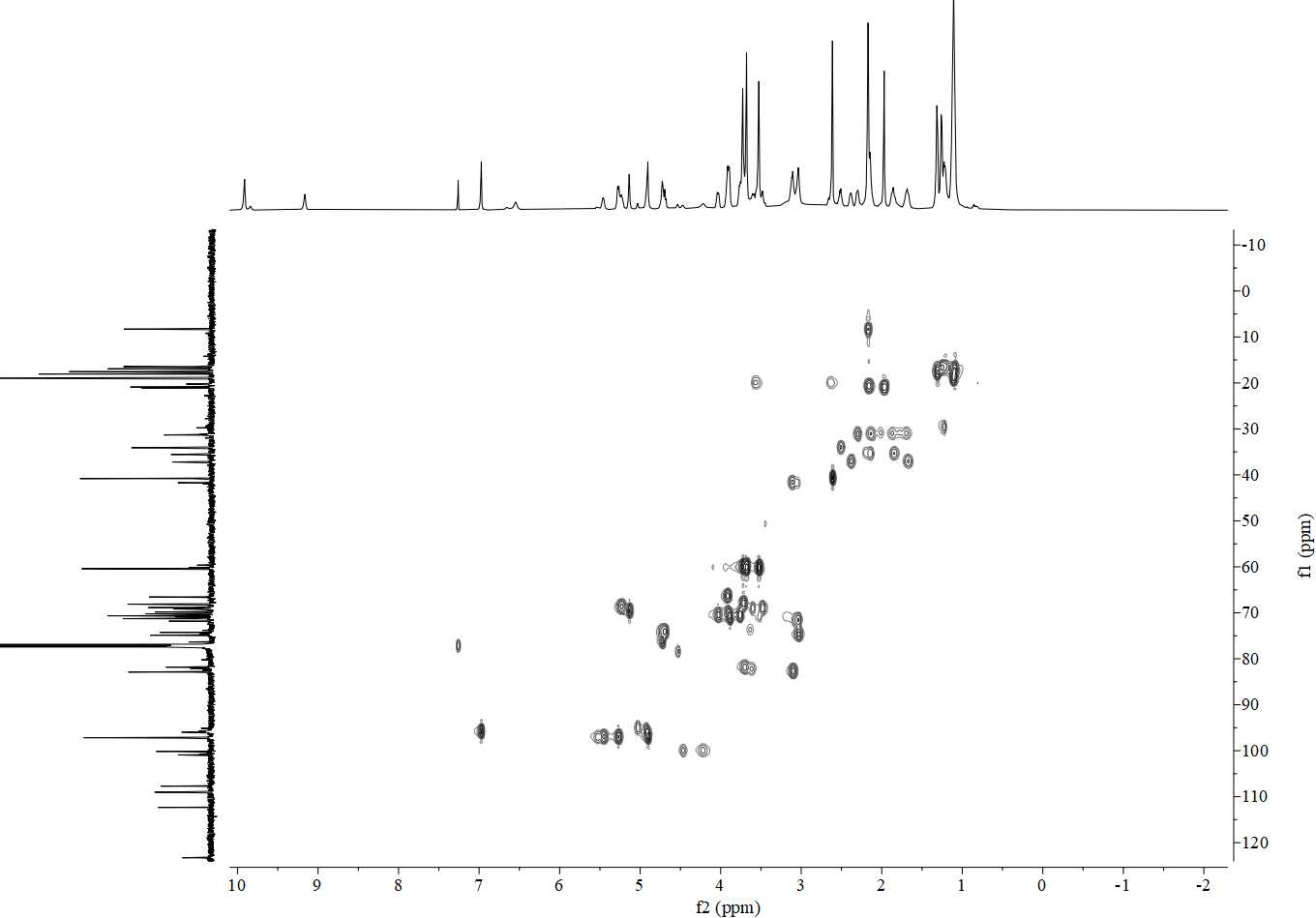


# (e)


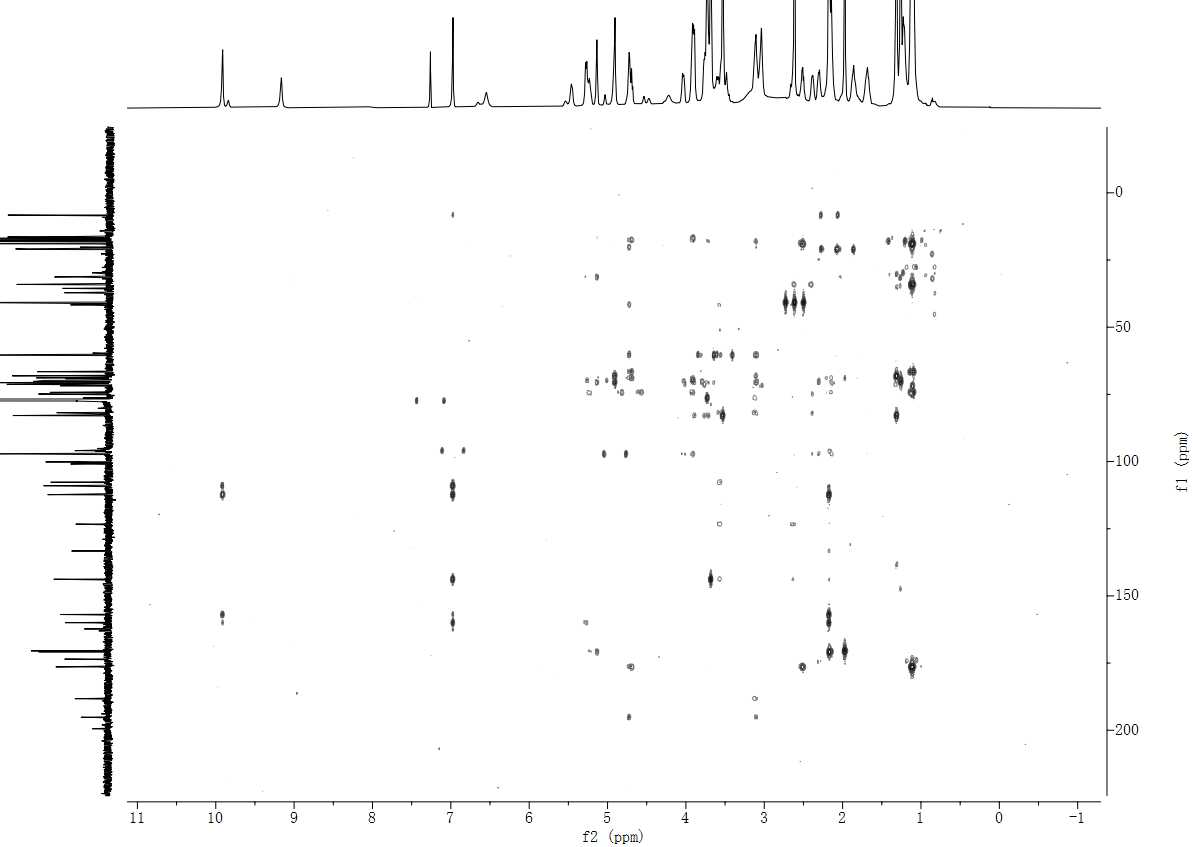


# (f)

_
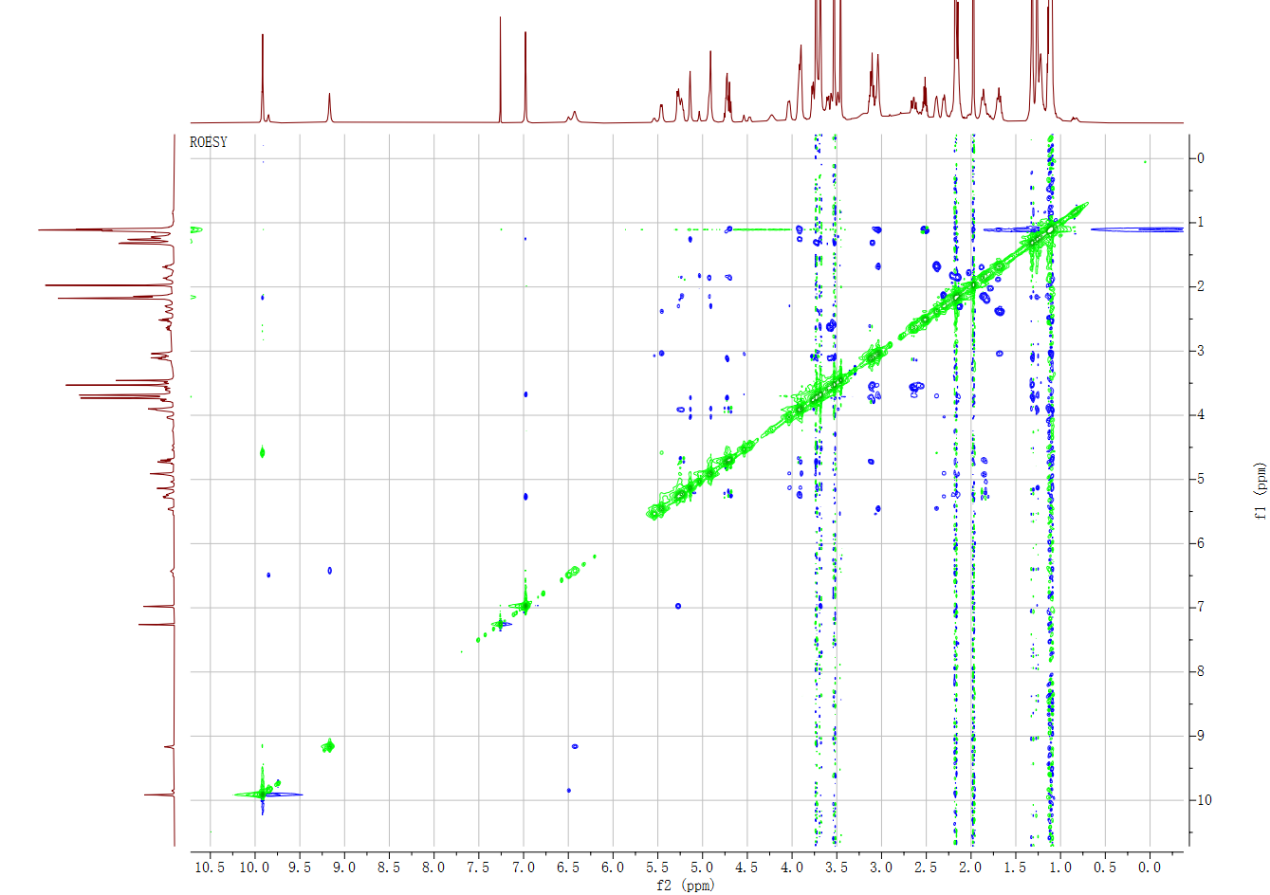
_

# Figure S5. NMR spectra of **1**. (a) ^1^H NMR of **1** (600 MHz, CDCl_3_). (b) ^13^C NMR of **1** (150 MHz, CDCl_3_). (c) ^1^H-^1^H COSY NMR of **1**_._ (d) HSQC NMR of **1**. (e) HMBC NMR of **1**. (f) NOESY NMR of **1**.

**Figure S6.**

**(a)**


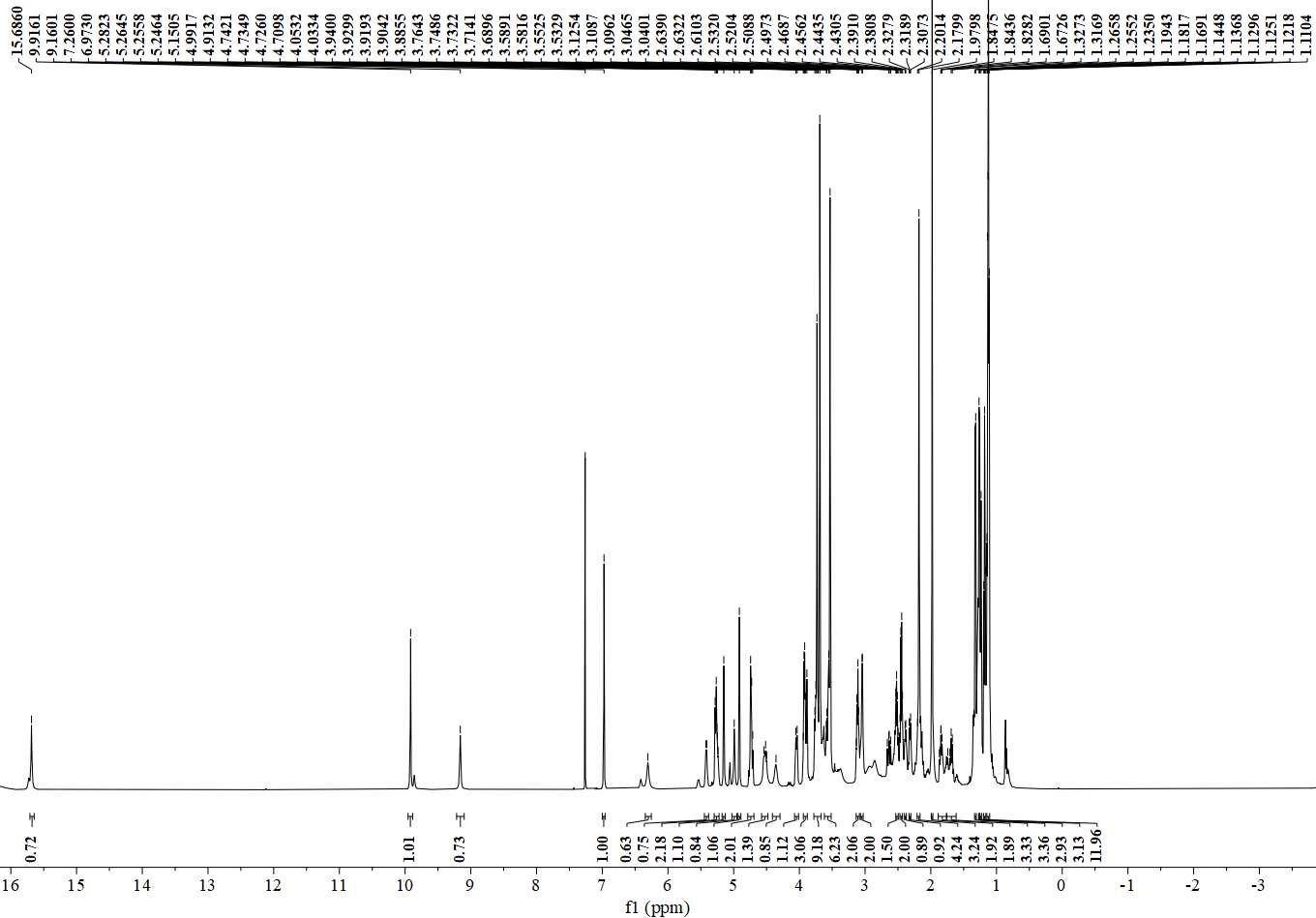


# (b)


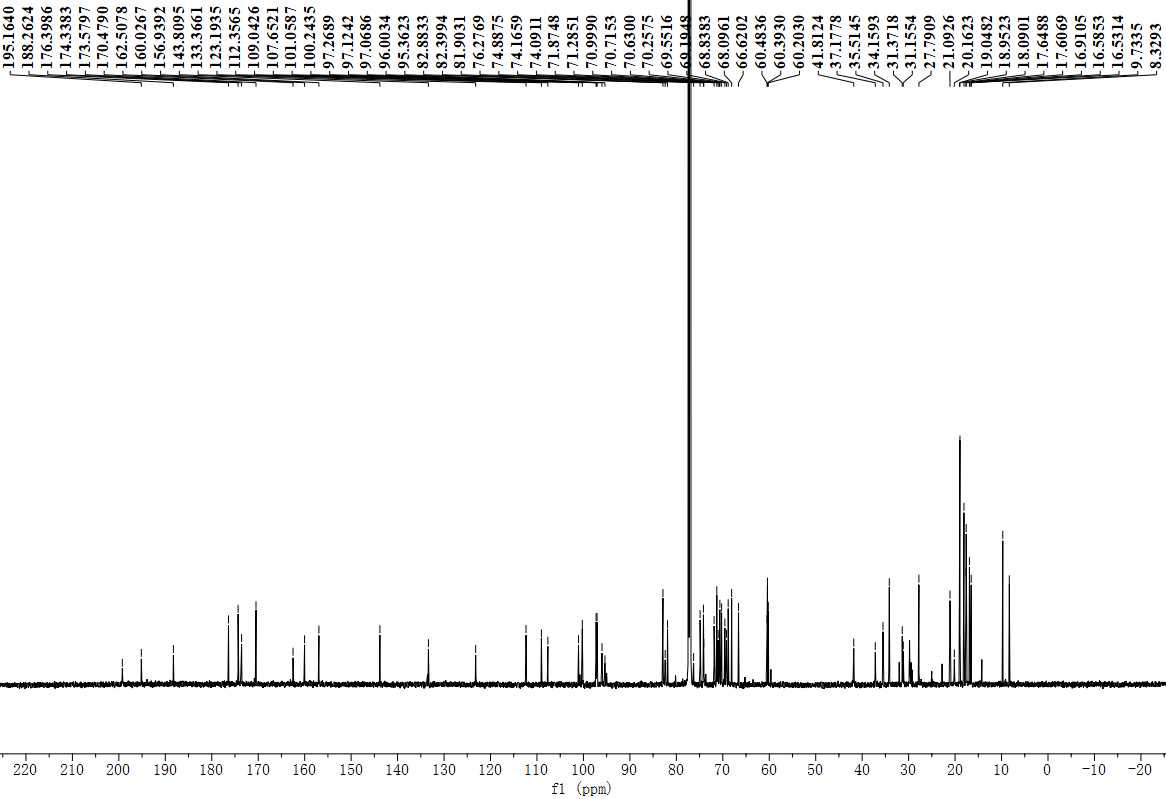


**(c)**


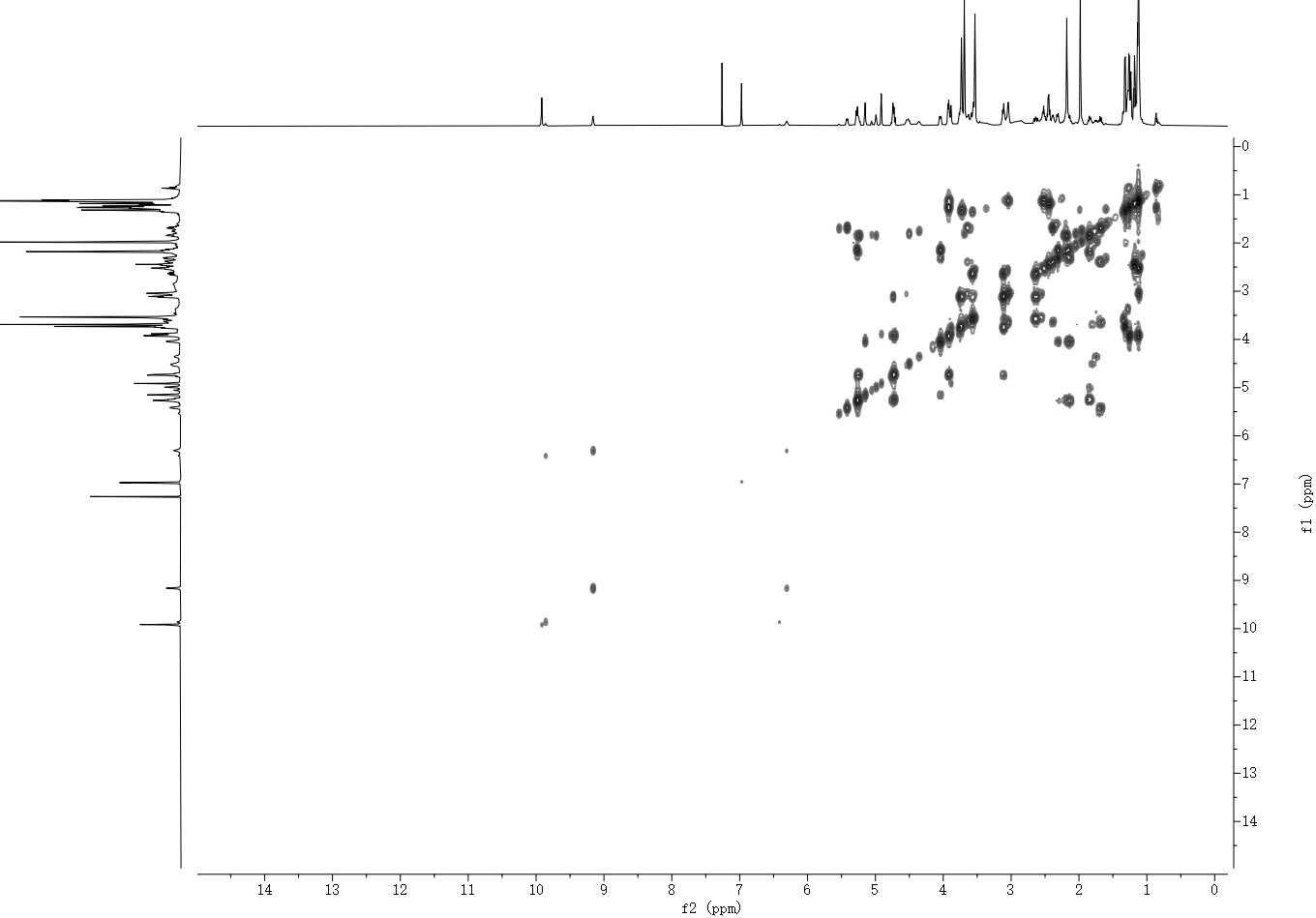


**(d)**


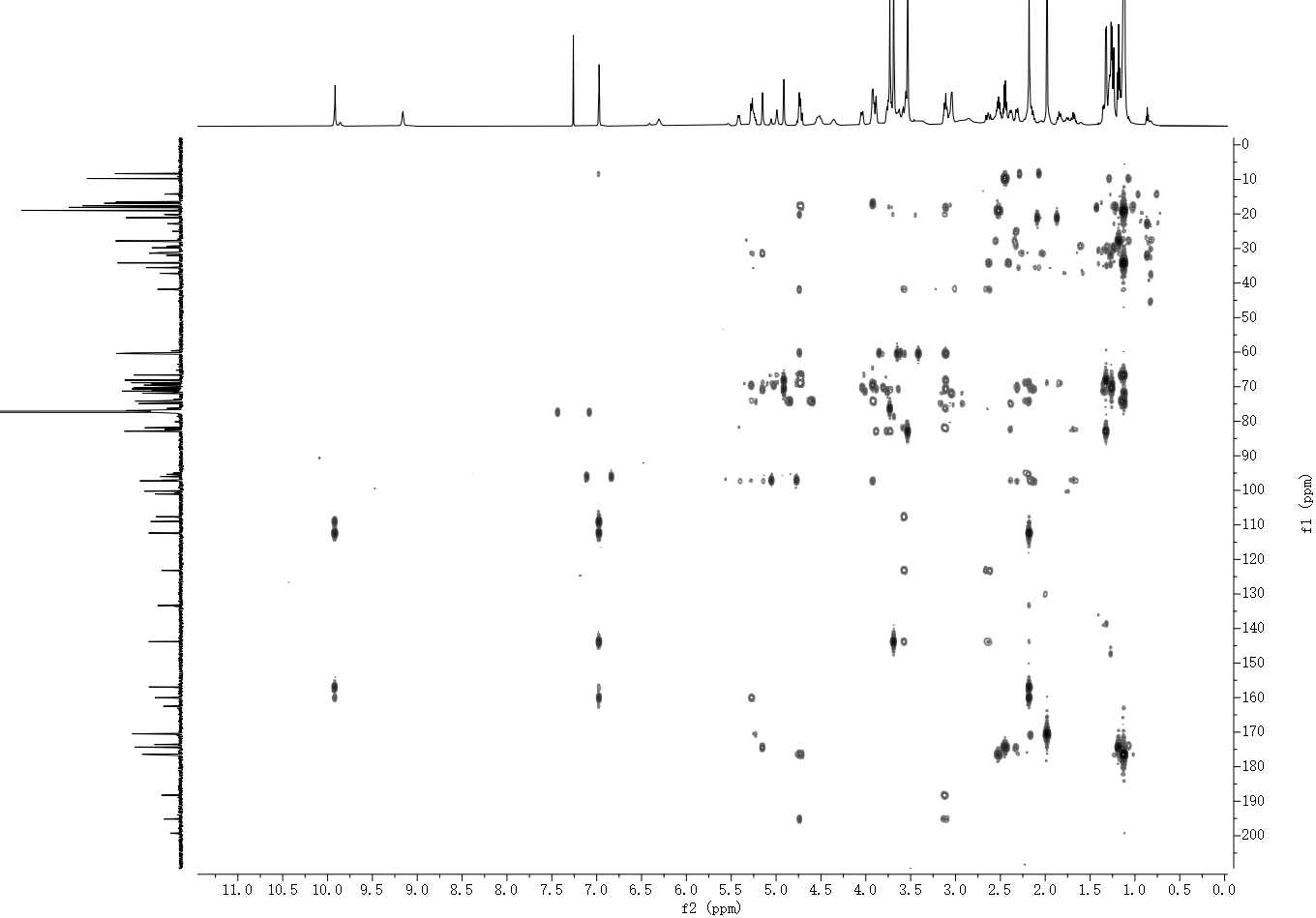


**(e)**


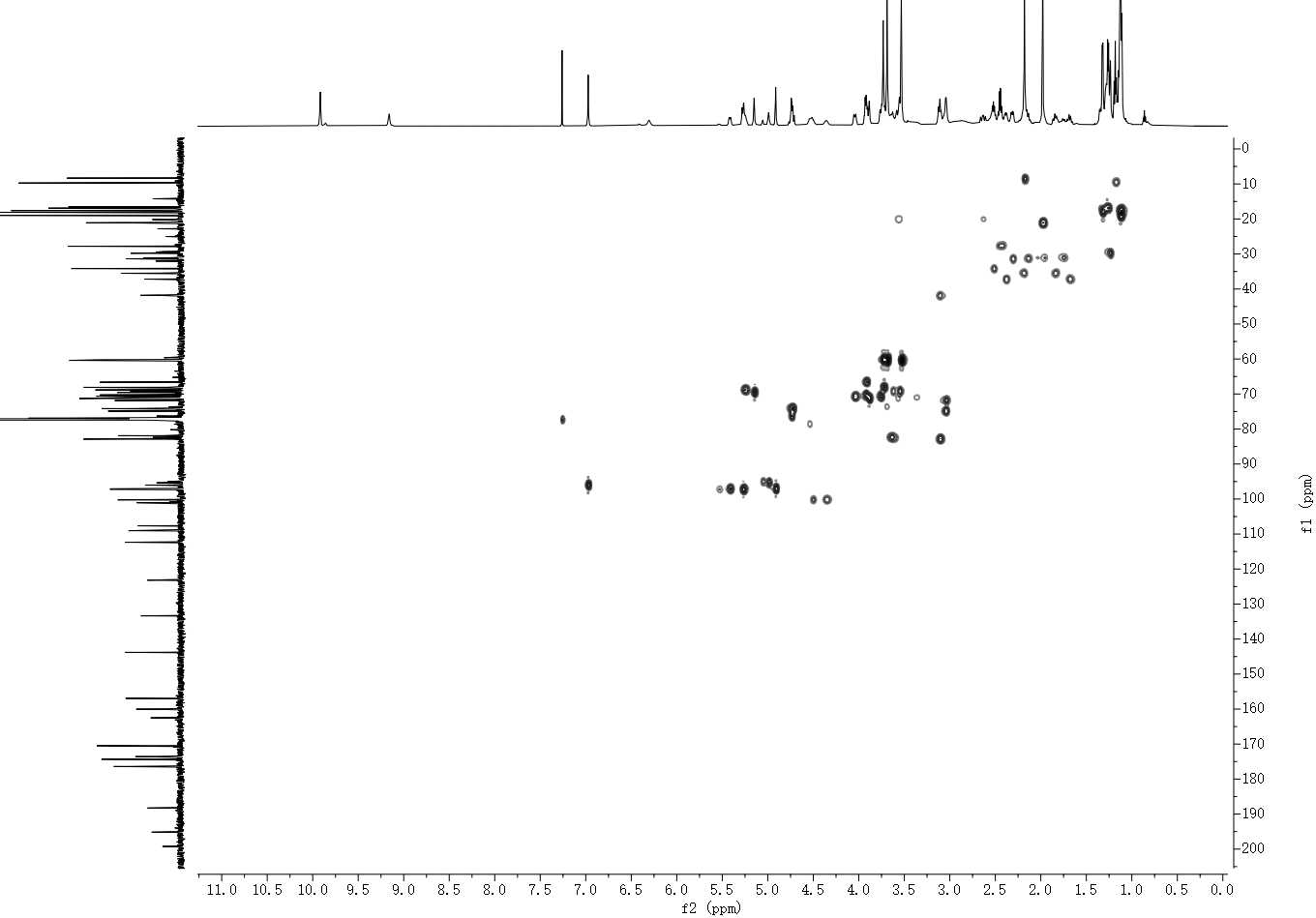


# Figure S6. NMR spectra of **2**. (a) ^1^H NMR of **2** (600 MHz, CDCl_3_). (b) ^13^C NMR of **2** (150 MHz, CDCl_3_). (c) ^1^H-^1^H COSY NMR of **2**_._ (d) HSQC NMR of **2**. (e) HMBC NMR of **2**.

**Figure S7**

# Figure S7. Structures of timmycin A and B.

**Figure S8**

**(a)**


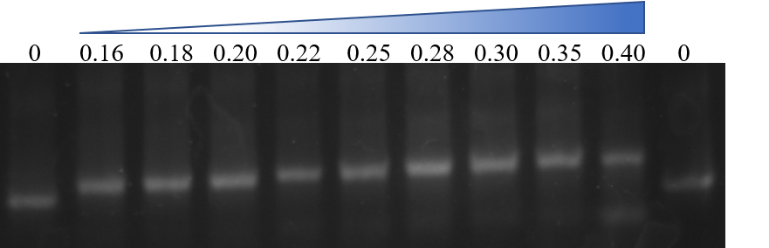


# **Figure S8**. Effect of **1** on the mobility of a 0.7 kb DNA fragment (from the genome of *Actinomadura* sp. ATCC 31491) on a 0.8% agarose gel. The different ratios of **1**/DNA are indicated at the top of each lane.

**Figure S9**


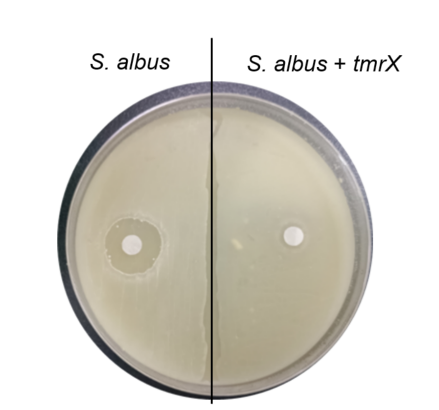


# **Figure S9**. *Streptomyces albus* J1074 expressing the UvrA-like protein TmrX from the *tim* BGC gains resistance to **1**. Note: the gene *tmrX* was cloned into a pSOK804-based integrative vector and introduced into *Streptomyces albus* J1074 through intergeneric conjugation. 0.25 μg of compound **1** was used for the disk diffusion test.
